# Tailoring Guidance in Internet-Based Interventions With Motive-Oriented Therapeutic Relationship

**DOI:** 10.3389/fdgth.2022.842487

**Published:** 2022-03-28

**Authors:** Anik Debrot, Laurent Berthoud, Franz Caspar, Thomas Berger, Valentino Pomini

**Affiliations:** ^1^Institute of Psychology, Faculty of Social and Political Sciences, University of Lausanne, Lausanne, Switzerland; ^2^Department of Clinical Psychology and Psychotherapy, University of Bern, Bern, Switzerland

**Keywords:** internet interventions, Motive-Oriented Therapeutic Relationship, guidance, tailoring, adherence

## Introduction

The feasibility and effectiveness of Internet interventions for mental health problems is well-established (1, 2). Most of the rapidly growing evidence comes from studies investigating guided self-help approaches, in which the presentation of a web-based self-help programme is combined with minimal but regular therapist contact. “Therapeutic guidance” often consists of a therapist's weekly scheduled written feedback per e-mail and the possibility for clients to ask questions (3, 4). Less common forms of guidance are “technical guidance” (5) or “guidance on demand” (6). The current literature suggests that unguided internet interventions are associated with lower adherence (7) and lower effects (8). However, guidance does not always seem necessary. For instance, for social anxiety disorders, good outcomes have emerged for unguided interventions if a proper diagnosis was established [e.g. (6, 9)]. Furthermore, the benefit of guidance in internet interventions for depression is evident only in moderately to severely depressed participants but not in mildly depressed individuals (8). Overall, recent evidence shows that the association of guidance with effectiveness is rather small (8) and that not all individuals need the same form and amount of guidance (3).

Little is known about how guidance should be personalized to improve adherence and outcome, and how to ensure optimal allocation of treatment resources (8). The Supportive Accountability Model (10) provides useful *general* guidelines on how to deliver guidance to *all* participants. It argues that human support enhances adherence in Internet interventions through accountability (i.e., the perception of a therapist being trustworthy, benevolent, and competent). However, to our knowledge, no theoretical framework specifies how to optimize *individualized* support. We argue that the Motive-Oriented Therapeutic Relationship (MOTR) approach, developed and investigated in face-to-face psychotherapy (11, 12), can be a promising avenue to tailor guidance, especially with participants experiencing difficulties with the self-help programs. Indeed, in the first controlled trial investigating MOTR in face-to-face therapy, there were fewer dropouts in the MOTR condition (13). Furthermore, adding an MOTR framework to a face-to-face therapy for borderline personality disorder yielded an additional reduction in general problems and a stronger therapeutic alliance (14). Next, we will introduce the MOTR approach with an illustrative example from a study on an internet-based guided self-help treatment for several anxiety disorders (15).

## Motive-Oriented Therapeutic Relationship

In guided internet-based self-help treatments, participants can struggle completing some tasks and exercises, as illustrated in the example below^1^, in this exchange of messages between a therapist and a participant. The female student, who suffered from panic disorder and depression, wrote the message below after using the program for 4 weeks. By then, she had already completed multiple exercises such as applied relaxation, cognitive restructuring with a thought record, and behavioral experiments [for a description of the programme; see (15)].

*Hi*,

*I'm feeling awful right now. I can't do what I wanted to do or what we discussed in the last message. It's just terrible! I didn't do the exercises in the program, I did not go shopping, and did not do anything for my studies. And my boyfriend says that's not so bad. Meanwhile, he complains about my whining. I am just demotivated, and everything is too much. I don't think I can do the exercises next week*. *Best, H*.

The therapist's response, which was not articulated according to MOTR, was:

*Dear H.*,

*Thank you very much for your message. You don't have to feel bad. It's not so bad that you did not manage to do the exercises. Your motivation problems will certainly pass. Please try the exercises anyway next week. Write me when you have done the activities. This also creates some commitment*. *I am looking forward to your email*. *Best wishes, D*.

Five minutes later, the participant answered:

*I feel totally misunderstood. I feel worse than ever, and you write to me that my motivation problems are passing. I don't just whine like that, and EVERYTHING IS TOO MUCH TO ME! YOU ARE LIKE MY BOYFRIEND!*

How can we understand the participant's reaction? The basis of MOTR is the so-called Plan Analysis (11, 12), a concept and method presuming that patients employ their behavior, especially their interpersonal behavior, to achieve certain goals, motives or needs. Plans consist of a purpose/motive and means that are serving them. They are organized hierarchically, whereby the highest level is represented by more universal, basic human psychological needs. Plans may be pursued both implicitly, unconsciously, and explicitly, in full consciousness.

What Plans might the participant pursue with her initial message? What does she want to achieve with her initial statement? When she states, “*I'm feeling awful right now*” and “*It's just terrible*,” she probably wants to show how badly she is doing and make sure that she and her problems are taken seriously. Furthermore, when she explains being “*demotivated*” and that “*everything is too much*” and adds “*I don't think I can do the exercises next week*,” she probably pursues the Plan to not be further overburdened with tasks.

What does the therapist do? Their behavior is not motive-oriented at all. Rather, by stating, “*You don't have to feel bad. It's not so bad that you did not manage to do the exercises. Your motivation problems will certainly pass*,” insinuating she will be able to do them later, they do not take her and her problems seriously. Moreover, with the request “*Please try the exercises anyway. Write to me when you have done the activities. This also creates some commitmen*t,” the therapist further overloads the participant. Consequently, the participant feels totally misunderstood and not taken seriously by the therapist, just as by her boyfriend.

Therapists using MOTR proactively adapt their behavior to the participant's motives. Relying on MOTR and the underlying motives of the participant outlined above, the therapist could have answered the following:

*Dear H.*,

[In order to respond to the acceptable motive “*Make sure that me and my problems are taken seriously*”]: *Thank you very much for your email. I have understood that you are very burdened, that everything is too much at the moment and I think it is good that you write to me so openly. While reading your message, I got the picture of a huge mountain of things that need to be done or that you want to do. And with the exercises and tasks, we have also contributed to this mountain*.

[In order to respond to the motive “avoid being overburdened by all these tasks”]: *What you have written to me is important, and you should continue to write to me in the future if I overwhelm you with the tasks. Can I count on you to keep writing to me immediately if I overwhelm you with an assignment? It is also crucial for next week that you avoid taking steps that are too big. “Small steps” are essential. Could you imagine thinking about what realistic small steps would be?* *Best wishes, D*.

As illustrated above, a principle of MOTR is to identify unproblematic motives that guide problematic behavior, and to proactively satisfy them. It aims to render behavior that is not useful for or even hindering therapeutic progress superfluous by providing the participants with what they need. Hence, MOTR aims to satisfy the motivational basis of “problematic” behavior that is not helping patients move forward. In our example, the participant first needed to do more to be taken seriously and avoid being further overburdened (“*EVERYTHING IS TOO MUCH TO ME*”) because the therapist did not recognize and satisfy the motives behind her behavior. By satisfying the guiding motives, the motivational basis of the complaining behavior weakens or dissolves, and the participant and therapist can focus on the actual therapeutic tasks at hand. For important additional considerations for MOTR, see Caspar (12).

## Applying MOTR to Internet Interventions

**Figure 1:**
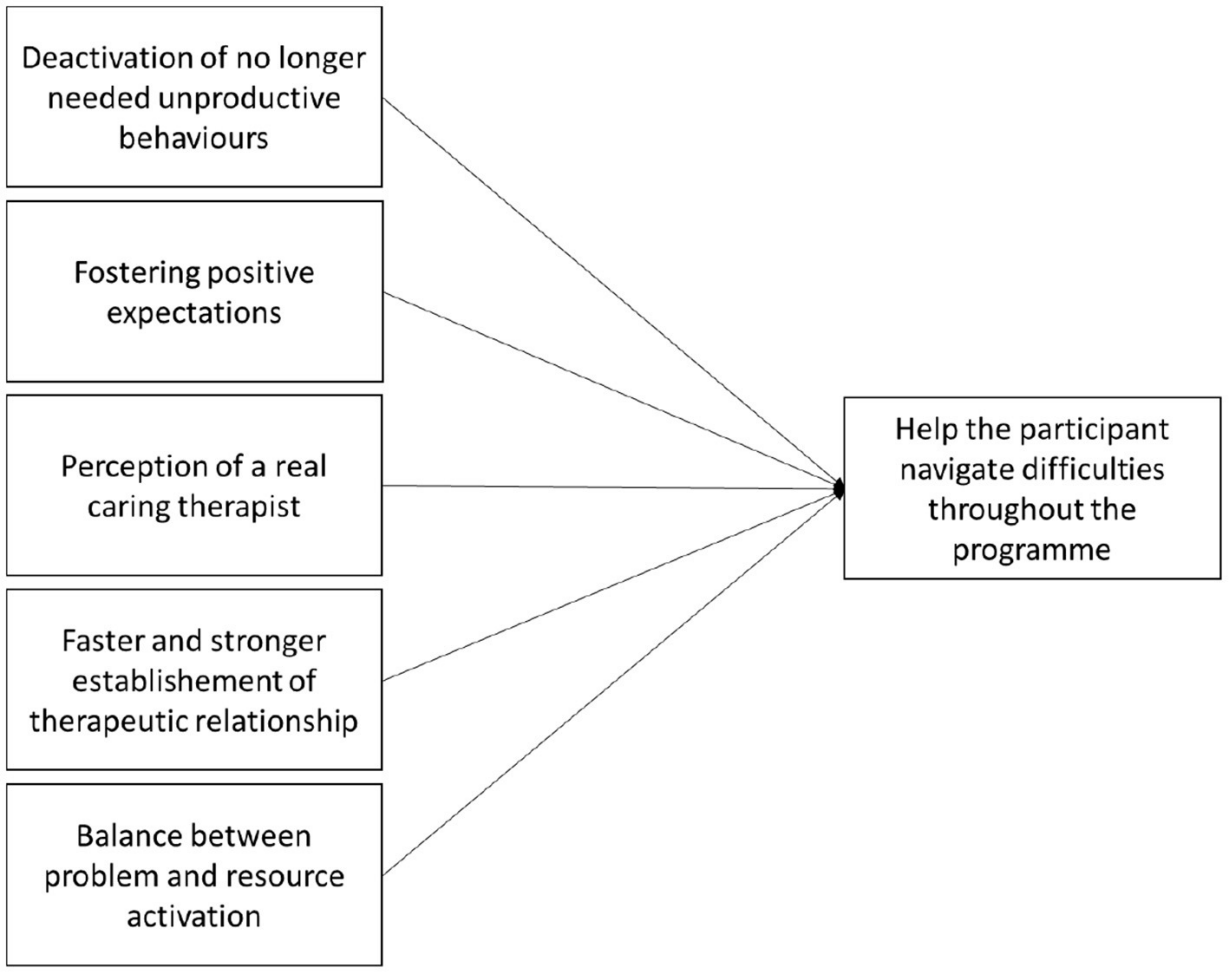
Expected MOTR processes in internet interventions.

The effects of MOTR on the quality of the therapeutic alliance and treatment outcome were demonstrated in several studies in face-to-face therapy [see (12)], including a randomized controlled trial with patients with borderline personality disorder (14). In guided internet-based interventions, there is only anecdotal evidence that the application of MOTR is feasible and useful (16). However, we can expect the application of MOTR within guidance to improve adherence and outcome of internet interventions (see Figure 1).

First, as outlined above, MOTR allows to focus on the tasks that help the participants reduce symptoms and improve their wellbeing. As illustrated above, participants no longer need unproductive behaviors when their motivational basis is satisfied.

Second, MOTR can foster patient's positive expectations toward the intervention. Indeed, with statements that are neither trivial nor trivializing, and that do not serve as an incentive which effect could be deleterious on a motivational level, MOTR can help to overcome moments of discouragement or rupture.

Third, individualized guidance allows participants to perceive a trustworthy and competent therapist on the other side of the screen, a genuinely caring human being able to understand their unique motives empathically. Thus, MOTR could increase the supportive accountability of therapists (10). Indeed, the significance of the therapist's credible communicated empathy for a successful therapeutic outcome was proven long ago (17).

Fourth, MOTR could speed up the establishment of a therapeutic alliance by making the therapist attuned faster to the participants' needs compared to conventional, somewhat standardized guidance. Current research suggests that the therapeutic alliance can also be established in internet interventions and is related to treatment outcomes (3, 18). However, this research often uses self-report measures. Because participants' expectations regarding the alliance in guided internet interventions usually are relatively low, the patients' alliance assessment is often very positive (3). Despite of this, there is room for improvement and deepening of the therapeutic relationship.

Finally, the tasks and exercises introduced in self-help programs, such as cognitive restructuring and exposure exercises, are often challenging for participants. Indeed, they often bring them into direct contact with painful emotions. In face-to-face therapy, evidence shows that productive therapeutic work is more likely if therapists do not mainly focus on problems and painful emotions, but also activate resources and focus on the patient's sound and healthy parts (19). In Internet interventions, resources activation is often realized in the guidance part. MOTR helps addresses a participant's individual motivational resources. Overall, MOTR can help to create a balance between problem and resource activation.

Hence, these five aspects allow to accompany the participant throughout different difficult moments during the intervention. At the beginning, some participants might not yet be in an action phase, ready to take steps to change and to realize the tasks and exercises delivered through the self-help program (20). They might thus benefit from the individualized motivational support provided by MOTR. During the intervention, participants may experience alliance ruptures, difficulties completing the exercises, understanding the psychoeducation, or seeing the meaning of their participation. As illustrated above, MOTR can provide individualized solutions that help participants seeing the benefits of the interventions. Finally, at the end of the intervention, MOTR might help the participants deal with the difficulties of terminating the contact with the therapist by fostering productive work with the self-management tasks at hand.

## Conclusion and Outlook

There is little work on how guidance can be optimally implemented in guided self-help approaches. To apply MOTR to Internet interventions, therapists would ideally perform a Plan Analysis-based case formulation. We would recommend that the professionals be trained psychologists who have received specific MOTR training and some supervision during their first guidance experiences. Some training is particularly important, as a limitation to applying MOTR to Internet interventions is the limited information about the patient (i.e., lack of non-verbal information or medical history). We anticipate MOTR to be particularly useful for more difficult patients [e.g., with higher symptom levels, see (8), or with personality disorders, see (21, 22); or bipolar disorder, (23)]. Moreover, MOTR could be particularly suitable to a guidance on demand format, as the quality rather than the quantity of guidance appears to influence outcome (4). An RCT comparing an already tested or newly developed Internet intervention with classic therapeutic guidance vs. MOTR-based guidance would allow to assess the added benefit of such an approach.

MOTR provides a promising avenue to improve the quality and impact of guidance in Internet interventions. MOTR-based messages can be expected to improve the efficiency of guidance as they respond more closely to the participants' needs and functioning. Future research should investigate whether this approach can indeed help improve the adherence and effectiveness of guided Internet interventions.

## Author Contributions

AD and LB drafted the paper. FC, TB, and VP revised the work. All authors contributed to generate the ideas for the present paper and provided approval of the version to be submitted.

## Funding

This study was supported by the SNSF grant 100014_182840/1 (AD and VP) and the Swiss Centre of Expertise in Life Course Research (LIVES).

## Conflict of Interest

The authors declare that the research was conducted in the absence of any commercial or financial relationships that could be construed as a potential conflict of interest.

## Publisher's Note

All claims expressed in this article are solely those of the authors and do not necessarily represent those of their affiliated organizations, or those of the publisher, the editors and the reviewers. Any product that may be evaluated in this article, or claim that may be made by its manufacturer, is not guaranteed or endorsed by the publisher.

## Footnotes

^1^It is a modified example of real messages from Berger et al. (15), representing a prototype e-mail of rather difficult exchanges with participants.
